# Synonymous codon usage pattern among the S, M, and L segments in Crimean-congo hemorrhagic fever virus

**DOI:** 10.6026/97320630017479

**Published:** 2021-04-30

**Authors:** Mallikarjun S Beelagi, SR Santosh Kumar, Uma Bharathi Indrabalan, Sharanagouda S Patil, Ashwini Prasad, KP Suresh, Shiva Prasad Kollur, Veeresh Santhebennur Jayappa, Siddappa B Kakkalameli, Chandrashekar Srinivasa, Prabhakarareddy Anapalli Venkataravana, Chandan Shivamallu

**Affiliations:** 1 Department of Biotechnology and Bioinformatics, Faculty of Life Sciences, JSS Academy of Higher Education & Research, Mysuru-570015, India; 2 ICAR-National Institute of Veterinary Epidemiology and Disease Informatics (NIVEDI), Yelahanka, Bengaluru-560064, India; 3 Department of Studies in Food Technology, Shivagangotri, Davangere University, Davangere Karnataka-577 007, India; 4 Department of Microbiology, Faculty of Life Sciences, JSS Academy of Higher Education & Research, Mysuru-570015, India; 5 Department of Sciences, Amrita School of Arts and Sciences, Mysuru, Amrita Vishwa Vidyapeetham, Karnataka - 570 026, India; 6 Department of Studies in Environmental Science, Shivagangotri, Davangere University, Davangere Karnataka-577 007, India; 7 Department of Studies in Botany, Davangere University, Shivagangotri, Davangere Karnataka - 577 007, India; 8 Department of Studies in Biotechnology, Davangere University, Shivagangotri, Davangere Karnataka-577 007, India

**Keywords:** CCHF virus, S, M, L segments, Homo sapiens, Codon usage bias, Mutational pressure, Natural selection, Host adaptation

## Abstract

Crimean-Congo hemorrhagic fever (CCHF) virus is one among the major zoonosis viral diseases that use the Hyalomma ticks as their transmission vector to cause viral infection to the human and mammalian community. The fatality of infectious is high across the world especially in Africa, Asia, Middle East, and Europe. This study regarding codon usage bias of S, M, and L segments of the CCHF virus pertaining to the host Homo sapiens, reveals in-depth information about the evolutionary characteristics of CCHFV. Relative Synonymous Codon Usage (RSCU), Effective number of codons (ENC) were calculated, to determine the codon usage pattern in each segment. Correlation analysis between Codon adaptation index (CAI), GRAVY (Hydrophobicity), AROMO (Aromaticity), and nucleotide composition revealed bias in the codon usage pattern. There was no strong codon bias found among any segments of the CCHF virus, indicating both the factors i.e., natural selection and mutational pressure shapes the codon usage bias.

## Background:

The tick-born Crimean-Congo hemorrhagic fever (CCHF) virus is the most widespread zoonosis disease-affecting human. Reports of the CCHF virus from the regions around the world have shown that increases in the number of patients and viral spread getting higher every year [1]. Infection can be transmitted from infected ticks bite, handling an infected animal, direct contact with infected animal's blood, and it can be nosocomial. The life cycle of the ticks has the potentiality to get infected at any stage of life, in various mammalian species, hence infectious disease remains asymptomatic even after the augment of the virus. An increase in the expansion of Hyalomma ticks around the different geographic, cycle of tick-vertebrate-tick infection has been called the most widespread tick-borne virus on the earth. Sanitisation and maintaining hygiene around the pet or an animal can be the first line of preventive measures to control the infection. The first-ever eruption of disease as a Crimean hemorrhagic fever was reported during 1944-1945 in Crimea region. The antigenic resemblances between the Congo virus and a Crimean hemorrhagic fever made them rename it as Crimean-congo hemorrhagic fever [2]. Crimean-Congo hemorrhagic fever virus (CCHFV) causes Crimean-Congo hemorrhagic fever, a tick-borne disease that causes haemorrhage and is found severely infecting the continents such as Africa, Asia, and Europe. The CCHF virus is a single-stranded negative sensed RNA that belongs to the Bunyaviridae family and a member of the genus Nairovirus. The virus structure is enveloped and has three negative sensed RNA genomes S, M, and L respectively. The S encodes nucleoprotein, M encodes glycoprotein and L encodes RNA-dependent RNA polymerase. Hyperanemia, dizziness, fever, headache, myalgia, and photophobia are some of the clinical indications of the CCHF virus [3]. 

The codon usage bias is the most preferable factor in the biological evolution of most organisms. Codon bias is always known as the choice of synonymous codons that are non-random for every different gene or genomes. Particular codon bias is specific to the taken organism and can be affected by GC content, gene lengths, and gene expression level. To understand the molecular mechanism of expression, and the consequence of long-term evolution on a genome, it is important to study the recognition of a distinct pattern of codons that possesses the distinct type of biological influences. Codon bias is the trendiest and widely acknowledged hypothetical analytic technique that describe codon usage bias is mutation-selection balance, determination of the codon usage exhibits the collective results of three evolutionary forces: genetic drift within a sample, natural selection, and mutational pressure. Overall, shuffle in GC and AT(U) pairs to cause nucleotide composition bias leads to mutational pressure, efficiency to maximize the production of protein by the preferred codons are known natural selection and eradication of codon changes among the generations as a result of emigration and immigration at the population level will lead to genetic drift [4]. The evolutionary process, an adaptation of the virus to the host, genetic drifts, selection, and mutation pressure are some of the information that can be obtained from codon usage patterns. The bias in the codon usage pattern may show variations in gene expression and protein synthesis efficiency. The viral-host adaptiveness affects the replication efficiency, virulency, synthesizing proteins, and survival of the virus is an extent of the bias in the codon usage pattern [9]. Several studies have suggested that mutational pressure is the main force for the establishment of a codon usage pattern [5,6,7,8]. In the current study, we have attempted to explain the codon usage bias of each segment of the CCHF virus using the various bioinformatics tools and R programming modules. Known data shows an occurrence of mutational pressure and natural selection in the CCHF virus [5]. But the strive of analysing the CCHF viral genome segment-wise has been employed with various methodologies to study the significant variations in codon usage pattern.

## Methods:

### Data Collection:

Nucleotide sequences are the major factor of the data collection. The complete CDSs nucleotide sequences of S, M, L segments of Homo sapiens host of CCHF virus were downloaded separately from NCBI Virus (https://www.ncbi.nlm.nih.gov/labs/virus/vssi/#/) database in FASTA format. Overall, 157 sequences were retrieved and analyzed. The coding sequences of each segment were aligned and edited individually with MEGA (Molecular Evolutionary Genetics Analysis) software [6].

### Overall Nucleotide Content Analysis:

Overall nucleotide content of each segment S, M, and L, which is a composition of A T G C, more specifically nucleotide at 3rd position of the codons (A3%, C3%, T3%, G3%), other entities like GC, GC1, GC2, GC3, and GC12 (mean values of G&C at the 1st and 2nd position of codons) were calculated using MEGA software. Mononucleotide and GC contents frequencies were calculated using R Studio programming software using the required external library "seqinR" [7-8].

### Relative Dinucleotide Abundance Analysis:

The relative dinucleotide abundance may take a role while predicting the codon usage indices; analysis is used to predict the organism's favourable dinucleotide. Totally there are 16 variable occurrences of dinucleotide is possible, the outline of the dinucleotide frequency specifies both mutational and selection pressure [9], and Relative Dinucleotide Abundance of three segments S, M, and L of CCHF virus were calculated using the method defined by Karlin and Burge [10].

PXY = FXY /(FX FY)

Where FX & FY are the frequency of individual nucleotide and dinucleotides are denoted by FXY in the same equation. As a conservative criterion, PXY > 1.23 is considered as high and PXY < 0.73 is low relative abundance [10]. Dinucleotide frequencies were calculated using R Studio programming software using the required external library "seqinR".

### Relative Synonymous Codon Usage (RSCU) Analysis:

RSCU method is described as the ratio of the observed to the expected value for a given amino acid. Amino acid frequency or the length of the sequence does not affect the RSCU values. The codon that achieves the more than 1.6 values are overrepresented, whereas codons that lie lesser than 0.6 are underrepresented and the codon values that fall between 1.6 and 0.6 are considered to be unbiased or randomly used. The RSCU values were calculated by the following formula:

RSCU = g_ij_ / Σ^i^_j_ g_ij_

Where gij denotes, an observed number of the ith codon for jth amino acid, that has ni types synonymous codons [11]. RSCU values of all segments S, M, L were obtained and visualized using R Studio programming software and "seqinR" library.

### Effective Number of Codons (ENC) Analysis:

An ENC evaluation reflects the deviation of codon from random selection. Commonly, Effective number of codon range between 20-61 [12]. The value 20 signifies an enormously biased in which only one codon is being used to code for each amino acid. Whereas value 61 indicates no bias and the codons have been used equally. If the ENC values are less than 45, are deemed to have moderately biased codon usage [9].

The ENC value was evaluated using the following formula:

ENC = 2+ 9/F2 + 1/F3 + 5/F4 + 5/F6 

Where Fi (i= 2,3, 4, 6) denotes the average Fi in the i- fold degenerate amino acid family. Where the Fi value is calculated using:

Fi = n Σij=1 ( nj /n)2 - 1/n-1 

Where n denotes the sum of observed codons for particular amino acid; nj denotes the sum of the observed jth codon for a particular amino acid. The ENC values of the S, M, L segments of the CCHF virus were calculated in R Studio programming software, "vhica" library [13]. To illustrate the relationship between an effective number of codons and GC3 (sum of G&C nucleotide at the third position) the ENC plot was generated. This method defines and quantifies codon usage bias of gene or genome, which is the finest overall method for estimating absolute synonymous codon usage. Whereas the formula to calculate the ENC values is [10][14]

ENC^expected^ =2 + S + (29/ S2 + (1-S))

Where S represents the GC3 (sum of G&C nucleotide at the third position) content. If the ENC values situate on the expected standard curve, it specifies that the codon usage be impacted by mutational pressure. Values below the standard curve indicate that the values are restricted by another factor i.e., natural selection.

### Neutrality Plot Analysis:

The neutrality plot analysis is used to determine the effect of mutational pressure and natural selection that influences the pattern of codon usage. The neutrality plot was illustrated using the GC3 values against the mean of GC12. If GC3 values are significant and closer to 1, mutational pressure plays a major role to build the codon usage pattern over natural selection. The regression slope is =0 then, natural selection plays a major role [12]. The same technique was carried out for each S, M, L segments of the CCHF virus by plotting the GC12 values against GC3 values. The regression line on the neutrality plot is indicative of the mutational pressure [15][14].

### Parity Rule 2 (PR2) plot Analysis:

A PR2 or Parity rule 2 analyses was done by plotting the GC bias on abscissa [G3/(G3+C3)] and AT bias [A3 / (A3+T3)] on the ordinate. The analysis usually reveals comparative magnitude between natural selection and mutation pressure based on the genome composition [7]. The origin for both axes will be 0.5 (X= 0.5, Y= 0.5). This suggests that A=T, G=C. points situating on the origin indicates no deviation between natural selection and mutational pressure.

### Codon Adaptive Index (CAI) Analysis:

Codon adaptive index (CAI) is a method to measure the level of expression gene based on the coding gene. The range of the CAI lies between 0 and 1. The highest relative adaptations were gained by the most frequent codon. The coding sequence that acquires the highest CAI values is more preferred over the lowest CAI values [11]. In the current study, Codon adaptive index values of each segment were calculated using DAMBE 7.0 software, considering the reference of Synonymous codon usage of H. sapiens [13].

### Average Hydrophobicity (GRAVY) and Aromaticity (AROMA):

 The GRAVY is the total amount of hydropathy values of the entire amino acid in a sequence divided by the number of residues. The average range of hydropathy range from -2.0 to +2.0, hydrophobicity of a protein were indicated by positive values, hydrophilicity was indicated by negative values. Aromaticity (AROMO) is the frequency value of aromatic amino acids, i.e., Trp, Tyr, and Phe in a sample amino acid sequence. The total GRAVY and AROMO values were calculated using the CodonW tool (CodonW download | SourceForge.net).

### Correlation analysis:

Correlation analysis was carried out for each segment separately utilizing the nucleotide composition of A, T, G, C, A3, T3, G3, C3, GC, GC1, GC2, GC3, and other factors such as ENC, CAI, GRAVY, AROMO using R Studio programming software with "corrgram" library [16].

## Results:

### Data collection:

The coding nucleotide sequences of each segment, S (n = 48, l = 3944bp), M (n= 57, l= 1687bp), and L (n= 52, l = 3945pb) of the CCHF virus were retrieved from the NCBI Virus database. The alignment of the nucleotide coding sequence of all segments, the estimation of nucleotide composition, and removal of stop codons from each sequence of all the segments were done using MEGA X (MUSCLE algorithm for alignment) [7].

### Nucleotide content analysis in S, M, L segments of CCHF virus:

**Figure 1:**
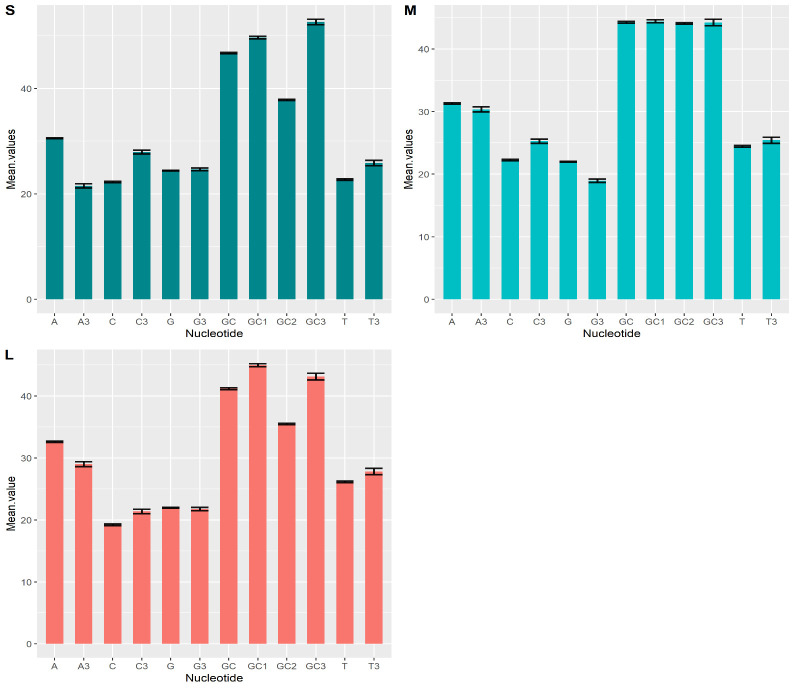
Graphical representation of total nucleotide composition of S, M, and L segments and error bars indicating standard deviation.

To determine the level of codon usage bias in each segment S, M, and L of the CCHF virus individually. The nucleotide compositions A, T, G, C & nucleotide composition at 3rd position, A3, T3, G3, C3, and G+C contents GC, GC1 (GC content at first codon position), GC2 (GC content at second codon position), GC3 (GC content at third codon position), values of S, M, and L segments of the CCHF virus are listed in (Supplementary Table). The nucleotide composition of each segment of the CCHF was calculated to assess the influence of nucleotide on codon usage patterns (Table 1 - see PDF). The evaluated nucleotide frequency values of each segment were as follow:

[1] Segment S: T (22.73% ± 0.42), C (22.26% ± 0.49), A (30.56 ± 0.36), G (24.43% ± 0.36), T3 (25.86% ± 1.19), C3 (27.95% ± 1.27), A3 (21.52% ± 0.87), G3 (24.65% ± 0.82), GC (46.70% ± 0.62), GC1 (49.66% ± 0.33), GC2 (37.83% ± 0.17) and GC3 composition was (52.60 ± 1.69). (Figure 1S)

[2] Segment M: T (24.22% ± 0.60), C (22.26% ± 0.53), A (31.31% ± 0.25), G (21.98% ± 0.26), T3 (25.42% ± 1.50), C3 (25.27% ± 1.09), A3 (30.34% ± 0.49), G3 (18.95% ± 0.81), GC (44.24% ± 0.68), GC1 (44.41% ± 0.54), GC2 (44.08% ± 0.55) and GC3 composition was (44.23% ± 1.72). (Figure 1M)

[3] Segment L: T (26.16% ± 0.14), C (19.22% ± 0.11), A (32.62% ± 0.11), G (21.27% ± 0.07), T3 (27.82% ± 0.49), C3 (21.38% ± 0.35), A3 (29.02% ± 0.39), G3 (21.76% ± 0.26), GC (41.20% ± 0.14), GC1 (44.98% ± 0.24), GC2 (35.22% ± 0.12) and GC3 composition was (43.15% ± 0.52). (Figure 1L)

### Relative Dinucleotide abundance frequency analysis:

The bias of dinucleotide can influence codon usage bias. Calculation of relative abundance of total 16 dinucleotides of each segment S, M, and L was calculated using R Studio software. The abundance frequency of each segment was seen to have less consistency compared with a theoretical value (equal to 1.0). Overall abundance frequency is classified based on overrepresented (>1.23) and underrepresented (< 0.78 ) [17] (Table 2 - see PDF).

**Figure 2:**
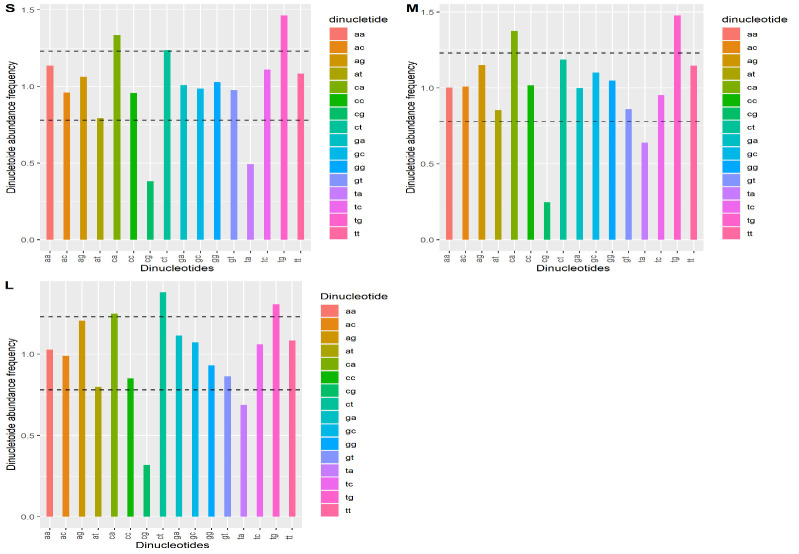
Distribution of Relative Dinucleotide abundance frequency of S, M and L segments of the CCHF virus, each colour represents different dinucleotide frequency, the line indicates over and under representation frequencies >1.23, <0.78, respectively.

[1] Segment S: Among the 16-dinucleotide bases, CA (1.33) and TG (1.46) were overrepresented whereas CG (0.38) and TA (0.49) were underrepresented (Figure 2S).

[2] Segment M: CA (1.37) and TG (1.47) dinucleotide were overrepresented; CG (0.24) was underrepresented (Figure 2M).

[3] Segment L: Dinucleotides CA (1.24), CT (1.37), and TG (1.30) were overrepresented; CG (0.31) is underrepresented (Figure 2L).

### Relative Synonymous Codon Usage (RSCU) Analysis:

**Figure 3:**
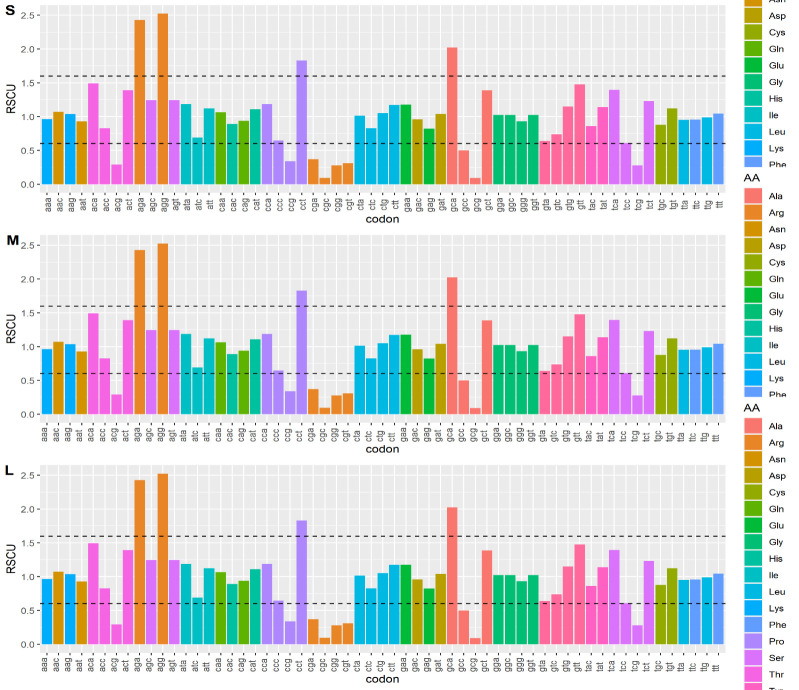
Bar graph representation of relative synonymous codon usage of S, M, and L segments. Lines on the graph indicate the over (>1.6) and underrepresented (<0.6).

The relative synonymous codons usage of each codon of the three segments was calculated. RSCU values are represented based on the range from 0.6 to 1.6. Values that are < 0.6 are considered as underrepresented and values > 1.6 are overrepresented. Codons that gain the significance value of >1.0 represent the positive codon bias and < 1.0 represent the negative codon bias [14][9] (Table 3 - see PDF). The result of the Relative Synonymous Codon Usage of each segment was as follows:

[1] Segment S: There were 7 codons (AGA, AGG, CCA, GCA, CTT, TAC, TCT) overrepresented, 9 (ACG, CAA, CCC, CCG, CGG, GTA, TAT, TCG, TTA) underrepresented, and 27 higher frequency codons, 24 lower frequency codons were observed. Under the higher frequency codons, the majority of the codons were terminated with nucleotide T (10 codons) and in the lower frequency codons, most of the codons were terminated with nucleotide A (9 codons). Figure 3S

[2] Segment M: segment M contains 6 (ACA, AGA, AGC, AGG, GCA, TCA) overrepresented, 9 (ACG, GCG, CCG, CGA, CGG, CTC, GCG, TCC, TCG) underrepresented, and 29 higher frequencies, 28 lower frequency codons were noticed. Most of the observed higher frequency codons were terminated with nucleotide A (9 codons) and lower frequency codons were terminated with nucleotide C (9 codons). Figure 3M

[3] Segment L: 4 (AGA, AGG, CCT, GCA) codons were overrepresented, 9 (ACG, CCG, CGA, CGC, CGG, CGT, GCC, GCG, TCG) underrepresented and 31 higher frequency codons, 28 lower frequency codons were noticed. Higher frequency codons were observed to have nucleotide T as dominant terminating nucleotide (14 codons) whereas lower frequency was seen to have a nucleotide G as a dominant terminating nucleotide (12 codons). Figure 3L

### Analysis of mutation pressure and natural selection on codon usage bias:

The analysis of ENC, PR2 bias, Neutrality of S, M, and L segments of the virus was performed to investigate the factors that impacting on the codon usage pattern. R Studio programming tool was used to calculate and analyse all parameters.

### Effective Number of Codons (ENC) analysis:

**Figure 4:**
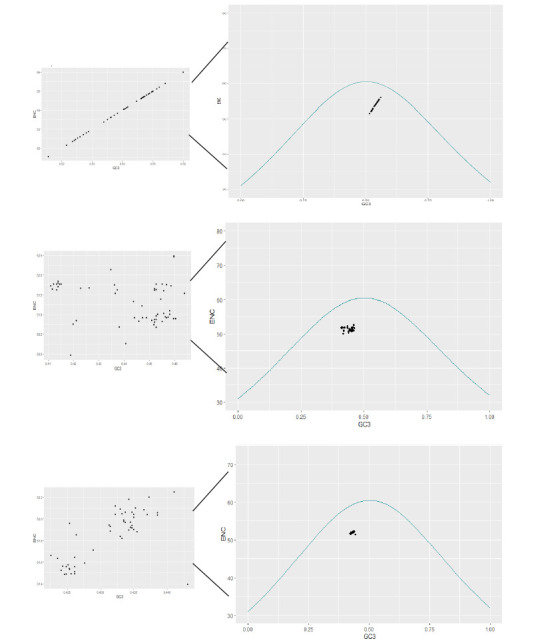
ENC plot – which illustrates the relationship between ENC values and GC at the 3rd position of each segment of the CCHF virus. The curve in the plot represents the standard expected codon usage.

Effective number of codon values was estimated to quantify the extent pattern of codon usage among each S, M, and L segments. The ENC values were varied from 51.55-56.00, 49.96-52.48, and 51.39-52.24 of S, M, and L segments, respectively. The mean value with a standard deviation of 54.4 ± 1.03, 51.30 ± 0.51, and 51.85 ± 0.24, respectively, Figure 4. An ENC-GC3 plot was illustrated to examine the role of mutational pressure among S, M, and L segments, results show that all points were situated below the standard curve, indicating the possibilities of mutational pressure (supplementary table).

### Parity rule 2 (PR2) plot analysis:

**Figure 5:**
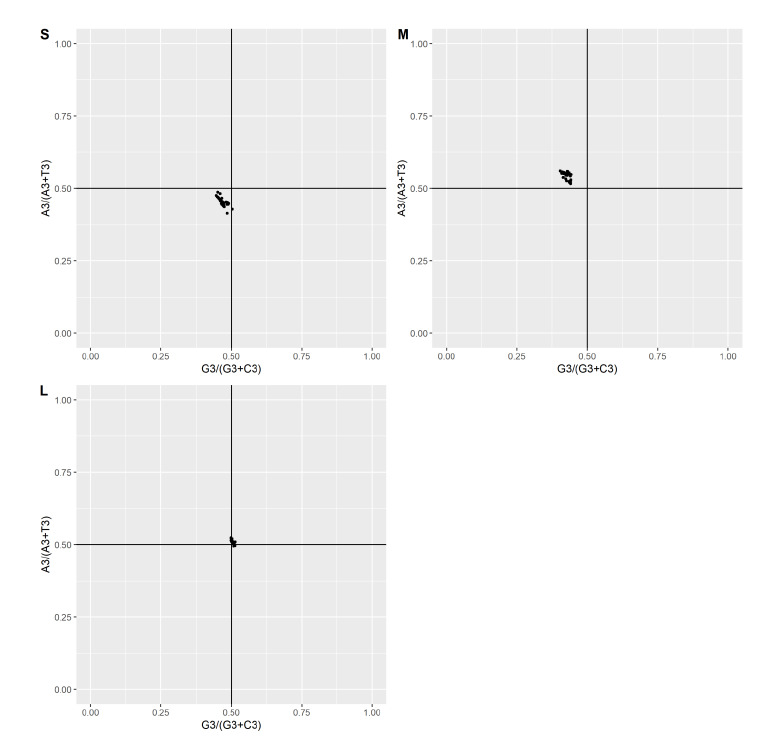
Parity Rule 2 bias plot of each segment of the CCHF virus, indicating the magnitude between natural selection and mutational pressure.

Parity rule 2 analyses was used to investigate the effect of selection and mutational pressure. The values of AT bias of 3rd position and GC bias of 3rd positions were used against each other to illustrate the PR2 plot. The X-ordinate represent [G3/(G3+C3)] and Y- represent the [A3/(A3+T3)]. The mean value of GC and AT bias of each segment is as follows:

[1] Segment S: Bias of GC and AT was 0.46 and 0.45, respectively. Suggesting the preference of pyrimidines over purines. Figure 5S

[2] Segment M: GC and AT bias of M segments were 0.42 and 0.54, respectively. Indicating the preference of AT over GC and purines over pyrimidines. Figure 5M

[3] Segment L: Whereas GC and AT bias of L segment was 0.50 and 0.51, suggesting the AT preference over GC, and purines over pyrimidines. Figure 5L

Figure 5 represents the parity rule 2 plot, in which 0.5 were the centre of both co-ordinates and the place where A≠T, G≠C. Values of GC bias and AT bias of the S & M segments were not equal to each other; hence the significant deviation and bias was observed. The points were situated at the upper left quadrant of the M segment and the bottom left quadrant of the S segment. Whereas deviation across some points of L segments was situated closer to 0.5 origins, indicating slight or low bias. PR2 analysis confirms that there is a bias at the 3rd position of GC and AT, indicating selection pressure over mutational in building the codon usage pattern.

### Neutrality plot analysis:

**Figure 6:**
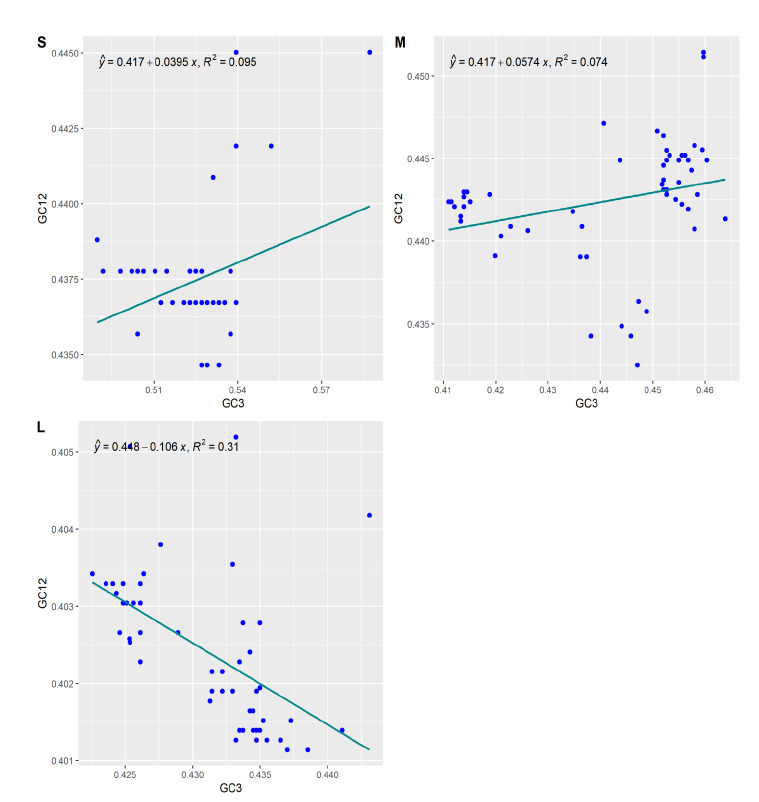
Neutrality plot: to analyze the impact of natural selection and mutational pressure on codon usage. GC12 on the Y-axis represents the Mean values of GC at first & second position, GC3 on the X-axis represent the frequency value of GC3, the line represents the regression line.

A neutrality plot is used to examine the relationship and dominant factors (mutational pressure and natural selection) between GC12 and GC3, the plot was illustrated using the mean values of the first and second position of GC against GC3. In this study, the neutrality analysis of S, M, and L segments was seen as follow:

[1] Segment S: Mean values of GC12 & GC3 were situated around the regression line and observed neutrality values were significant positive regression between GC12 & GC3 with y = 0.417+0.0395, R2 = 0.095, the importance of neutrality is 3.95%. thus the natural selection plays a major role compared to mutational pressure in shaping the CUB (codon usage bias). Figure 6S

[2] Segment M: Mean values of GC12 & GC3 were situated closer to the regression line and the positive significant regression line was seen with the value of y = 0.417+0.057, R2 = 0.074, and showing 5.5% importance of neutrality. CUB of M segments is also influenced by natural selection over the mutational pressure. The amount of genetic disparity within the population is determined by the rate of mutation. And these disparities arose from the errors made during the replication process. Figure 6M

[3] Segment L: values of GC12 and GC3 were situated near negative significant negative regression line with y= 0.448-0.106, +100x, R2= 0.31. Highlighting the 10.6% neutrality. Natural selection plays a major effecting factor in CUB. Figure 6L

### Codon Adaptation index analysis:

The Codon adaptation index was executed to examine the optimization of codon usage and adaptation of the virus to the host. CAI values were calculated by considering the codon usage pattern of H. sapiens as a reference. This study identified that all segments of CCHF possess a higher tendency of CAI values (> 0.5). The CAI values were varied from 0.75 to 0.77, 0.71 to 0.74, 0.71 to 0.72 with a mean value ± standard deviation of 0.76 ± 0.007, 0.73 ± 0.008, and 0.71 ± 0.002 in S, M, and L, respectively.

### Correlation analysis:

**Figure 7:**
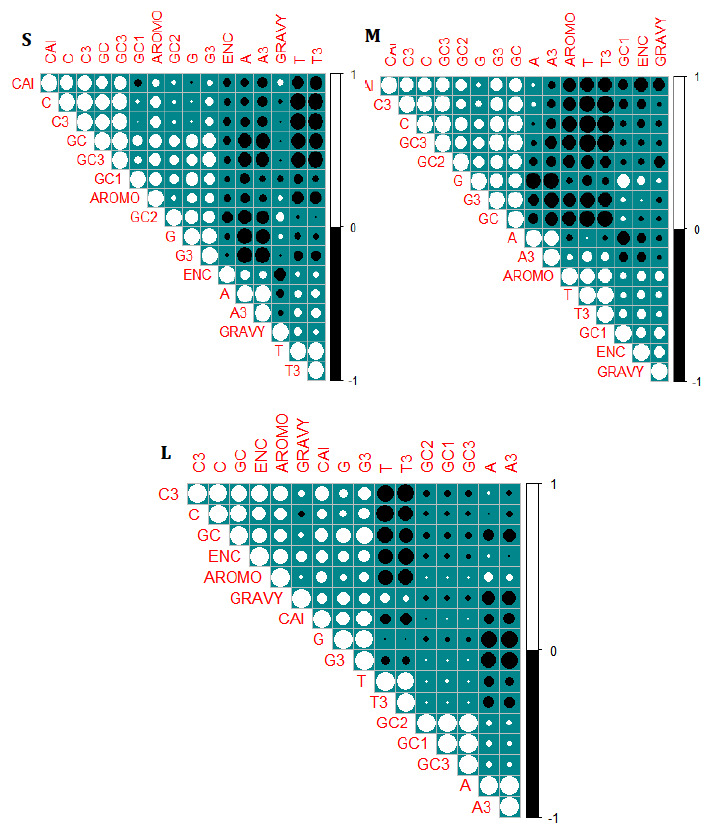
Graphical representation of Correlation of each segment of the CCHF virus. Solid black color indicates the negative correlation, and white represents a positive correlation between the variables.

The major two determinants, natural selection, and mutational pressure were considered to study the codon usage bias in each segment of CCHF. To further confirm the natural selection, the correlation analysis was performed among T, C, A, G, GC, GC1, GC2, GC3, ENC, CAI, GRAVY, and AROMO. The significant values r= -0.21675, and r = 0.4764 were observed between the ENC and GC3 of the S and L segment respectively, indicating that the pattern of codon bias is influenced by GC nucleotide on the third position. Whereas in the M segment of the CCHF virus, a non-significant value r = -0.1945 was obtained. A significant correlation was seen between CAI and GC3 r = 0.6528, r = 0.7138 of S, and M segments, respectively. Also, indicates that the influence of GC on the third position impacts the CUB. But the correlation value r = 0.0020 of segment L of the CCHF virus has a non-significance value, saying non-impact of GC3 on CUB. The correlation between CAI and ENC was significant value r= -0.29674, r=-0.675, and r= 0.476 observed between S, and M, and L segments, respectively. The correlation between ENC & GC3 were non-significant value r = -0.21675, r = -0.19458, and r = -0.08070 seen among S, M, and L, respectively. Suggesting that, GC3 alone does not affect the CUB of the CCHF virus. Significant correlation values between ENC & AROMO r = -0.23729, r = 0.35052 of M, and, L were observed, but non-significant values r = 0.66411 were seen in the S segment, indicates that the effect of Aromaticity presence in M and L segment and absent in S segment. Significant correlation between ENC & GRAVY r = -0.54394, r = 0.49216, and r = 0.34495 seen among S, M, and L segment, respectively. The effect of hydrophobicity is present in all segments of the CCHF. Correlation between the rest of the nucleotide compositions was observed as in (supplementary Table.2), and Figure 7S,Figure 7M,Figure 7L

## Discussion:

CCHF is zoonotic, tick-borne, and one among the virus affecting on the human community. In the majority of living organisms, the choice of particular codon usage is a major sign of biological evolution. Therefore, the codon usage pattern delivers significant information about the host adaptation, evolution, and factor influencing CUB [5]. We suggest that the segmentation of the entire genome is a molecular key that reduces the communication between capsid stability and geometrically constrained viral particles. So, analysing the genome segment-wise will lead to identifying the specific residual differences in the genes of the viral genome.

The nucleotide composition is the base element to shape the codon usage pattern. Interestingly, the mean value of nucleotide A and A-end codons was seen to be the highest in each segment of the CCHF virus. Also found that selection of nucleotide A was consistent even when the entire nucleotide of the genome was studied as in previous findings [5]. The high nucleotide content of A in the CCHF CDs may be a genomic feature of genus Nairovirus. The CA & TG and CG & TA dinucleotides were seen to be over and underrepresented, respectively in S, M segments whereas in the L segment CT dinucleotide was an addition to overrepresented codons. Similar results were observed in the previous study [9,15]. Further, a total of 7 (ACG, CCG, TCG, TGC, AGC, GGC, and TGC), 11(ACG, CCG, CGC, GCG, TCG, AGC, CGC, GCA, GCG, and TGC), and 10 (AGC, CGC, GCA, GCG, GGC, TGC, CCG, CGC, GCG, and TCG) RSCU codons were containing underrepresented CG dinucleotide in S, M, and L segment respectively, signifying all these codons are not favourably preferred. so, this study says that dinucleotide composition performs an impact on codon usage pattern. Also, we attempted to track the shape of the codon usage bias of each segment in the CCHF virus i.e., S, M, and L. The previous CUB study of the entire genome of the CCHF was able to determine 31 high-frequency RSCU codons from the entire genome but when we analyzed the genome by each different segment, we have achieved to track 27, 29, and 31 high-frequency codons in S, M, and L segment, respectively.

The previous study states that the lower ENC values cause high-level gene expression & codon usage [9,16]. To calculate overall codon usage bias, we calculated ENC values of each segment of the CCHF varied from 51.56 to 56 (average of 54.07 ± 1.03), 49.96 to 52.48 (average of 51.30 ± 0.51), and 51.39 to 52.24 (average of 51.85 ± 0.24) were the observed among S, M, and L segments, respectively, indicating the low or weak codon usage bias favour effective replication in a host cell with a different preference in codon usage. A similar result was observed in a previous study as well [9,16]. The previous analysis of CAI of the CCHF virus shows that the entire CAI was observed to be 0.80, but compared to this study, observed maximum CAI values of each S, M, and L were 0.77, 0.74, and 0.72 respectively, which signifies the low adaptation ability to the host compared to a previous study [5]. Similar results were also found in the CUB of the Rift Valley fever virus that belongs to the same family Bunyariridea [20].

The magnitude between natural selection and mutational pressure wasn’t seen in the previous study [5], in order to analyze, we performed parity rule 2 analysis. The obtained results reveal that segment L has a slight or low bias compared to S, and M. Also, purines over pyrimidines in M and L segment whereas pyrimidine over purines in S segments. The parity plot of the GC and AT on the third position revealed that natural selection making a remarkable role over mutational pressure. A neutrality analysis plot was performed to determine the influencing factor on CUB, based on the previous study, when the entire genome was analysed, there was no significant relationship observed between GC12 and GC3 [5]. But analyzing the genome segment-wise reveals the importance of neutrality 3.95%, 5.5%, and 10.6% in S, M, and L segments, respectively. An indication of a natural selection over the mutational pressure in effecting the codon usage bias [21]. In the earlier study, only the correspondence analysis was performed segment-wise and all the other analyses were carried out for S, M, and L segments combined with H. sapiens, Hyalomma, Bos taurus, and Ovis aries organism. Parity rule 2 was employed in this study which also determines the influence of mutation pressure and natural selection in analysing the codon usage bias, which was not analysed in the previous study [5]. However, analyzing the entire genome segment-wise, resulted in significant interpretation compared to the previous study, it was observed that there are distinct variations while studying the entire genome compared to analysing the genome segments-wise. Further, the RSCU and ENC values among the segments S, M, and L indicated that S was dominating over the segments M and L. Natural selection makes a significant role in building the codon usage pattern when the gene is highly expressed, meanwhile mutational drifts play a major role when there is an occurrence of low-level gene expression. Based on these two factors, the origin of codon usage can be clarified. But it seems that these two factors aren't sufficient enough to confirm attributes of codon usage [22]. The disease-related wet-lab experiments are needed for confirmation about the codon usage pattern but with time constraints the codon usage bias analytical technique has been a boon approach to predict and analyze the CUB computationally.

## Conclusion:

The CCHF virus is one of the deadliest viral diseases that causes a major public health concern. Indeed, there are no potential medicines available, so there is a need for the development of potential drugs and therapeutic. Studying the potential host and viral genome may be beneficial in identifying various preventive measures. We examined that, the CCHF virus had a weak codon usage bias, and adaptation of the CCHF virus to the host varies from each segment, meanwhile this study proves that the virus has less adaptation capability with humans. Also, the RSCU, ENC, CAI, Aromaticity, Gravy, and other nucleotide bases play a different role in each segment of the CCHF virus to undergo natural selection and shaping the codon usage pattern, respectively. The results of the study will aid future CCHF surveillance and other basic research that provides significant insights into the understanding of CCHF evolution.
